# Analysis of longitudinal data of Nellore cattle from performance test at pasture using random regression model

**DOI:** 10.1186/2193-1801-1-49

**Published:** 2012-11-20

**Authors:** Fernando Brito Lopes, Cláudio Ulhôa Magnabosco, Fernanda Paulini, Marcelo Corrêa da Silva, Eliane Sayuri Miyagi, Raysildo Barbosa Lôbo

**Affiliations:** 1 Embrapa Cerrado / Capes, Road GO-462, km 12, Rural Area, PO Box 179, CEP: 75375-000 Santo Antônio de Goiás, Brazil; 2 Embrapa Cerrado/CNPq, Brasília, DF, Brazil; 3 Program in Animal Biology, Universidade de Brasília, Brasília, DF, Brazil; 4 Program in Animal Science, Federal University of Goiás, Goiânia, GO Brazil; 5 Brazilian Society of Breeders and Researchers, Ribeirão Preto, São Paulo, Brazil

**Keywords:** Animal breeding, Beef cattle, Heritability, Zebu

## Abstract

This study was carried out to estimate (co)variance components and genetic parameters for live weight of Nellore cattle from Performance Test of Young Bulls using random regression models. Data of weights and ages of 925 weaned males was used. The animal model included the fixed effect of contemporary group, age of the animal at weighing as a covariate and as random effects it was considered the effect of additive genetic and permanent environment of the animal. The residue was modeled considering four classes of variances. The models were compared based on the Bayesian information criteria of Akaike and Schwartz. The model polynomial of fourth and sixth order for the direct additive genetic effects and permanent environment of the animal, respectively was the most appropriate to describe the changes in the variances of the weights during the period in which the animals participating in the performance test young bulls. Heritability estimates showed moderate magnitudes and indicated that direct selection will promote improvement of selection criteria adopted. Furthermore, due to high positive correlation between the estimated weights, it was suggested selecting the best animals before at 365 days of age, because it is the period in which the animals have a higher growth rate and thus you can select animals heavier and less delayed.

## Introduction

The beef cattle production has an important role for the Brazilian agribusiness. This production goes to increase around of 4.4% until 2015. This is possible due to great potential of Brazilian herd production, which is first in world exports and second largest producer of beef cattle and third in world food consumption (Brasil, 2011).

In Brazil, the Zebu breeds are more than 80% of national bovine herd. But, these animals have presented difference in growth and production in each Brazilian region due to diversity of climate and management system (Ferraz & Eler, 2010). Many researchers have studied growth traits as weight in different ages, growth curves and growth rate traits of Nellore cattle in different regions of Brazil (Souza et al., 2008; Lopes et al., 2011; Lopes et al., 2012; Santos et al., 2012). However, research that evaluates the growth and development of young bulls in performance test are incipient. Mainly research that use random regression models, which consider all weights of the animals and not just standardized weights to specific ages. (Meyer, 2001; Albuquerque & Meyer, 2001; Sousa Jr. et al. 2010; Boligon et al., 2010; Baldi et al., 2012; Selapa et al., 2012).

In accordance to Macedo et al. (2009), the covariance functions and random regression models have been considered as an alternative for the adjustment of records obtained in sequence from the same animal along time and which presents a structured pattern of covariance. This occurs due to the fact that phenotypic traits can be measured using same animal for a large number of times. In performance test, many traits are measured for a large number of times. In these tests, the animals have the same average age, are of the same sex, same race, same feed management and are in the same environment and climate. So, it is possible more easily identify the best animals. Therefore, the essential aim of performance test is to help in the identification of genetically superior animals.

The main objective of breeding programs is the selection of genetically superior animals, based on selection criteria specific to each program. However, the performance of future progeny does not depend solely on parental genotypes, but also the environment and genotype × environment interaction. Thus, there is no way to remove the influence of environmental on the animal performance. In the performance test the animals should be contemporaneous, created on the same management and feeding. Therefore, there is the effect of genotype x environment interaction and correlation between genetic and phenotypic differences can be maximized.

Thus, this study was carried out to estimate (co)variance components and genetic parameters for live weight of Nellore cattle from Performance Test of Young Bulls using random regression models.

## Material and methods

Data set used in this study was composed by 5,550 records of weight-age of 925 Nellore cattle from Performance Test of Young Bulls, whit general mean and standard-deviation of 294.14±60.80. A summary of the data is given in Table 1. These animals were raised at pasture in Capivara Farm of the Embrapa Rice and Bean during the period of 2001 to 2012.

**Table 1:** **Summary of the data**

Feature	Number
Number of sires	371
Number of sires with progeny in the data	245
Number of dams	478
Number of dams with progeny in the data	245
Number of animals with records	925
Number of animals in pedigree	4,293
Mean age (days)	409
Starting age range (days)	209–597
Contemporary groups	133
Mean initial body weight (kg)	147
Mean final body weight (kg)	506

This farm is located in Santo Antônio de Goiás City, Goiás State, latitude of 16°28´00”S, longitude of 49°17´00”W and altitude of 823 m. In accordance with Köppen this city is classified as Aw climate, savanna tropical and megathermic. The mean air temperature is 23.0°C and the minimum and maximum temperature occurs in July and September with average temperature of 14.2°C and 31.7°C, respectively. It present two distinct season, rainy season, between October and April; and dry season, between May and September. The annual average precipitation is 1,485 mm and the annual average of relative humidity is 71%. The soil is dystrophic red latosol and clayey and your environment is savanna biome and relief plan.

**Figure 1:**
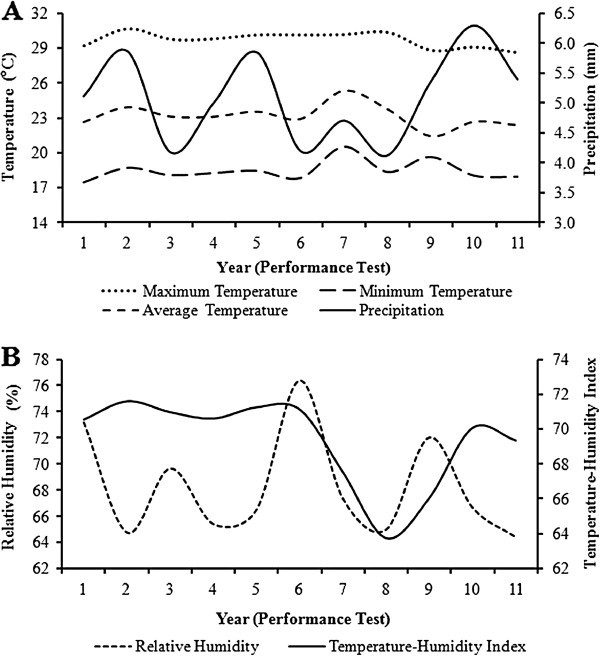
**Average**, **minimum and maximum temperature and precipitation (A)and relative humidity (%)and temperature and humidity index (B)obtained during the Performance Testing of Young Bulls(TDTJ)raised at pasture.**

The annual distribution of average, minimum and maximum temperature (°C), precipitation (mm), relative humidity (%) and temperature humidity index were collected in Climatological Station of Embrapa Rice and Bean, during the years 2001 to 2012 are presented in Figure 1.

In the performance test the animals were fed with *Brachiaria brizantha*, cv Marandu and cv Xaraés, in rotational grazing (9 days of occupation by 36 days of rest) defined in accordance with forage offer. It was supplied 7 kg of forage to each 100 kg of life weight (LW). Thus, the animals can to select the good forage during the test and were fed with mineral and proteic supplement of minimum consumption during de dry season. Average weight and age at the beginning of the test was 200 kg and 8 to 10 months, respectively. Animals were weighed regularly during the period of 294 days of performance test.

The animals’ weights of performance test were analyzed using random regression models. It was considered as fixed effect the contemporary groups (animals that born in the same year and season and were weighed on the same day) and age of animals as covariate adjusted using Legendre polynomials of third order. As random effects it was considered the direct additive genetic effect and animal environment permanent. This model was:

Where, *y*_*ij*_ is the *i*^*th*^ measure of *j*^*th*^ animal; *FE* is the set fixed effect; b_*m*_ is the regression coeficient; *ϕ*_*m*_(*t*_*i*_) = Legendre polynomial regression function; *ϕ*_*m*_(*t*_*ij*_) = Legendre polynomial regression function of each animal *j* for age ,(*t*_*i*_) in accordance with additive direct genetic effect and animal environment permanent; *α*_*jm*_ and *δ*_*jm*_= random regression coefficients for additive genetic and permanent environment effects, respectively, for each animal; *k*_*b*_, *k*_*A*_ and *k*_*C*_ = Legendre polynomial order; *e*_*ij*_= random error associated with each age *i* of animal *j*. The random regression model in its matrix form is:

Where E[y] = Xβ, E[α] = 0, E[β] = 0 and E[e] = 0 and (co)variance matrix was:

Where, *y* is the measure vector; β is the fixed effects vector (FE and *b*_*m*_); α is the random coefficients vector of direct addictive genetic effects; δ = coefficient vector of animal permanent environment; *X*, *Z*_1_ and *W*_1_is the matrix of fixed effect, direct addictive genetic and animal permanent environment effects, respectively; *K*_*A*_ and *K*_*C*_ is the (co)variance matrix of direct addictive genetic and animal permanent environment effects, respectively; *A* is the relationship matrix; *I* is the identity matrix; *N*_*α*_ = number of animals with valid records; *R* is the residual variance matrix; and *e* is the error vector.

The *Wombat* device (Meyer, 2007) was used for the data analysis. Among the many available algorithms of this device, the PX-AI algorithm was chosen, because, it combines the PX (parameter expanded) method, which is a variation of the EM (expectation maximization) algorithm, with the AI (average information) algorithm (Meyer, 1997). Eighteen models with Legendre orthogonal polynomials of first to sixth order were used to describe the effects of direct additive genetic and animal permanent environment of the animal. It was used four residual variance class in according to the following ranges of age: 209 ≤ *t* ≤ 299 days, 300 ≤ *t* ≤ 399 days, 400 ≤ *t* ≤ 499 days y 500 ≤ *t* ≤ 597 days, where t is the animal age.

Selection of models was based on Akaike’s information criterion (Akaike, 1974). Akaike (1974 proposed a simple and useful criterion (AIC) for selecting the best-fit model among alternative models: AIC = −2 log (maximum likelihood) + 2 (number of model parameters). Differences between AIC values are important, not the absolute size of AIC values. The model with the lowest AIC is considered the best. Various experiences verify the applicability of AIC in model selection (Wada & Kashiwagi, 1990; Burnham & Anderson, 1998). Another widely used information criterion is the Bayesian Schwarz information criterion (BIC), which takes into account model uncertainty as well Schwarz, (1978). The Bayesian Schwarz information criterion is stricter than the AIC. The BIC is defined as BIC = −2 log (maximum likelihood) − log (n) × (number of model parameters), where n is equal to the number of records used in the analysis (Burnham and Anderson, 1998).

## Results and discussion

In accordance with Akaike and Bayesian information criteria the M46 model showed the best fit (Table 2). The convergence was hampered when there was increase in coefficients of the polynomial, which was also reported by many authors (Meyer, 1999; Arango et al., 2004; Boligon et al., 2010). Dias et al. (2006) showed that while increase the order of polynomials there is also increase on flexibility of curve. However, when higher was order of polynomials higher will be computational resources and worse will be the convergence (Kirkpatrick et al., 1994; Meyer, 1998).

**Table 2:** **Order of fit of additive direct** (**K**_**A**_), **animal permanent environment effects** (**K**_**C**_), **number of parameters** (**Np**), **log**-**likelihood value** (**log**), **Akaike information criterion** (**AIC**) **and Bayesian information criterion** (**BIC**)

Model	K_A_	K_C_	Np	log	AIC	BIC
M31	3	1	11	−17502.758	17513.758	17550.040
M32	3	2	13	−17500.159	17513.159	17556.038
M33	3	3	16	−17481.499	17497.499	17550.273
M34	3	4	20	−17440.644	17460.644	17526.611
M35	3	5	25	−17412.969	17437.969	17520.428
M36	3	6	31	−14666.907	14697.907	14800.157
M41	4	1	15	−17467.977	17482.977	17532.453
M42	4	2	17	−17464.705	17481.705	17537.777
M43	4	3	20	−17449.091	17469.091	17535.058
M44	4	4	24	−17436.942	17460.942	17540.103
M45	4	5	29	−17410.104	17431.104	17514.757
**M46**	**4**	**6**	**35**	−**14243**.**361**	**14278**.**361**	**14393**.**804**
M51	5	1	20	−17444.606	17464.606	17530.574
M52	5	2	22	−17441.162	17463.162	17535.727
M53	5	3	25	−17424.647	17449.647	17532.106
M54	5	4	29	−17417.428	17446.428	17542.081
M55	5	5	34	−17408.834	17442.834	17554.979
M56	5	6	40	−17405.868	17445.868	17577.803

**Figure 2:**
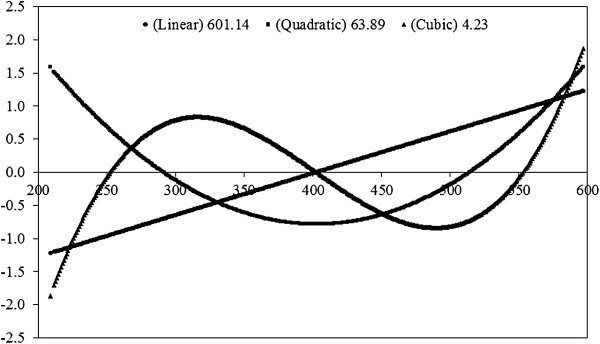
**Genetic direct eigenfunctions(linear, quadratic, and cubic)and respective eigenvalues(646**.**05, 159.84, and 41.08)at different ages estimated using M46 models.**

The three main eigenfunctions for additive direct effects obtained with model M46 are shown in Figure 2. Eigenfunctions are continuous functions whose coefficients are formed by the elements of the eigenvectors of the (co)variance matrices. A specific eigenvalue is attributed to each eigenfunction and corresponds to the proportion of total variation that is explained by such eigenfunction. Eigenvalues and eigenfunctions of genetic covariance functions provide an insight into the way selection affects the character under consideration (Kirkpatrick et al., 1990).

The three main eigenvalues of the additive direct (co)variance matrix explained 100% of total additive variance. The first eigenvalue accounted for 89.82%. The first eigenfunction was negative down of 400 days and positive up to 400 days of age; but the linear trend indicates a positive correlation between weights. Thus, selection for greater weights at any age will increase other weights. There was decrease of second eigenfunction up to 400 days of age and increased thereafter. This change indicates that selection for this component will have opposite effects at the beginning and at the end of the trajectory. However, the genetic change due to selection for this component would be small because it was responsible for only 9.55% of additive variance. Similar results were reported by Albuquerque & Meyer (2001) for growth from birth to 630 days of age in Nellore cattle.

Estimates of (co)variance between the random regression coefficients for M46 model are showed in Table 3. It was observed that the intercept showed the major variance for the direct addictive genetic effect. The estimates of correlation between the intercept and liner regression coefficient was medium magnitude (0.38), 0.68 between the intercept and quadratic coefficient and −0.17 between the intercept and cubic coefficient. For the animal permanent environment all correlation estimated were negative. Similar results were reported by Meyer (2001), Albuquerque & Meyer (2001),Sousa Jr. et al. (2010), Boligon et al. (2010) and Baldi et al. (2012).

**Table 3:** **Estimates of variance** (**diagonal**), **covariance** (**below the diagonal**) **and genetic correlation** (**above the diagonal**) **between the random regression coefficients and eigenvalues** (**λ**) **of the** (**co**)**variance matrix for the direct genetic effects and animal permanent environment**

Effect	Order of fit	1	2	3	4	5	6	λ
Additive direct	1	586.46	0.38	0.68	−0.17			601.14
	2	79.33	74.08	−0.11	−0.54			63.89
	3	39.83	−2.28	5.80	−0.43			4.23
	4	−7.19	−7.88	−1.77	2.92			0.00
Animal permanent environment	1	638.09	−0.04	−0.18	−0.40	−0.47	−0.06	646.05
	2	−9.77	108.68	0.60	0.44	0.46	−0.11	159.84
	3	−41.38	56.28	81.85	0.88	0.76	0.07	41.08
	4	−40.00	18.35	31.73	15.80	0.82	−0.07	7.84
	5	−29.76	11.96	17.19	8.21	6.29	−0.48	1.72
	6	−3.59	−2.84	1.55	−0.70	−2.90	5.82	0,00

**Figure 3:**
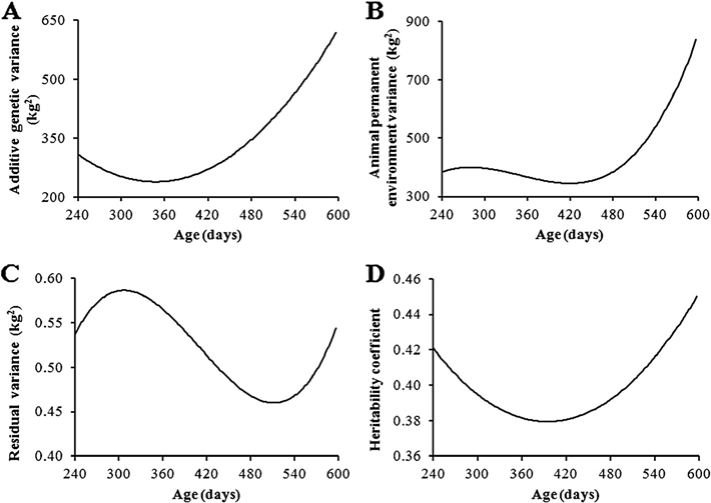
**Direct additive genetic variance curves (A), animal permanent environment variance curves (B), residual variance curves (C) and heritability coefficient curves (D) at different ages estimated using M46 models.**

The variance components and heritability coeficients estimated for animals weight are show in the Figure 3. There was decrease in adictive genetic variance between 240 and 360d, after this period there was increase of theses estimatives, corroborating the results of Boligon et al. (2010), Sousa Jr. et al., (2010). There was small variability in the variance componente of animal permanent environment between 240 days and 450 days of age. After 450 days there was high increase in this componente. In general, these results were corroborated by several authors (Albuquerque & Meyer, 2001; Nobre et al., 2003; Dias et al., 2006, Baldi et al., 2012; Selapa et al., 2012).

There was quadratic trend for heritability estimates with major values at beginning and end of performance test. The estimatives of heritability ranged from 0.35 to 0.46. Similar results were reported by Souza et al. (2008) which showed heritabilities of 0.15 until 0.45 between birth and 660 days of age. Boligon et al. (2010) showed range of heritability of 0.34 to 0.42 between birth and 550 days of age.

Estimative of variance components and heritability coefficients for weight at 240, 365, 450 and 550 days of age are showed in Table 4. It was observed major values of variances components and heritabilities at 240 and 550 days of age. The direct genetic correlation estimates between weights from 240 to 550 are shown in Table 5. The genetic correlation estimates decreased when distance between records increased.

**Table 4:** **Variance components and heritability coefficients estimates for different ages obtained through the Legendre order four models**

Age				***h***^***2***^
240	313.98	392.62	727.16	0.43
365	232.00	355.60	627.58	0.37
450	308.48	407.14	803.66	0.38
550	495.28	465.93	1073.13	0.46

: additive direct genetic variance; : animal permanent environment effect variance; : phenotypic variance; h^2^: heritability for direct effect.

**Table 5:** **Components of genetic covariance** (**below diagonal**) **and genetic correlation coefficients** (**above diagonal**) **estimates for different ages obtained through the Legendre order four models**

Age	240	365	450	550
240		0.90	0.75	0.75
365	241.72		0.95	0.90
450	233.06	252.91		0.99
550	233.06	304.34	387.87	

The genetic correlations were greater than 0.70 at most ages. Similar estimates, varying from 0.43 to 0.88, were described by Boligon et al. (2009). These results suggest that selection for greater weight at any age should increase mature weights (Boligon et al., 2010). The later the selection is performed, the greater the response in mature weight. Selection is performed using weight from weaning to 550 days of age, mainly. This might increase the adult size of animals, which is not always desired (Dias et al., 2006; Boligon et al., 2008). Arango et al. (2004) also reported strong genetic correlations between weight from 840 to 2,160 days of age (close to 0.84) and between 2,160 and 3,090 days of age (close to 0.90). Due to high genetic correlation estimated between these weights we suggested the selection of young animals, but those that had a higher growth rate, thus avoiding the selection of animal very heavy and late growth.

## Conclusions

The model that polynomials of fourth and sixth order for the direct additive genetic and animal permanent environment effects, respectively, were the most appropriate to describe the changes in the variances of the weights during the period in which the animals participating in the performance test young bulls.

Heritability estimates showed moderate magnitudes and indicated that direct selection will promote improvements in the selection criteria adopted. Furthermore, due to the high positive genetic correlation, we suggest selecting the best animals in the previous 365 days of age, because this is the period in which the animals have a higher growth rate and thus it can select precocious and heavier animals.
